# Oxidative Stress and Obesity- and Type 2 Diabetes-Induced Heart Failure

**DOI:** 10.3390/antiox9080653

**Published:** 2020-07-23

**Authors:** Luc Demaison

**Affiliations:** Unité de Nutrition Humaine, INRA, UNH, Université Clermont Auvergne, CRNH Auvergne, 63000 Clermont-Ferrand, France; luc.demaison@inrae.fr

Obesity is a risk factor for the development of type 2 diabetes (T2D), which is associated with cardiovascular diseases [1]. Although moderate obesity improves the function of cardiac mitochondria in animals [2] and humans (BMI = 32) [3], diabetes mellitus strongly deteriorates the activity of these organelles [4] and precipitates the myocardium toward heart failure [5]. The deleterious action of T2D on mitochondrial function seems to result from increased oxidative stress [6]. In contrast, moderate obesity triggers a reduction in oxidative stress [7] and an improved cardiac mechanical function [8].

A good strategy to treat heart failure in T2D consists of supplying adequate antioxidants [9]. However, antioxidants are numerous and each of them has specific properties. The choice of a good antioxidant is thus crucial.

This Special Issue, concerning T2D, heart function and oxidative stress, contains seven contributions, six research articles and one review, and it details recent advances on this topic.

In a first set of studies, the session describes the involvement of several enzymes in the oxidative stress observed in T2D. Steyn et al. [10] show the relationship between central obesity and plasma glutathione peroxidase-3 activity in men and women and explain the higher cardio-renal risk in women in terms of higher obesity status and tougher oxidative stress. In another study [11], the involvement of NADPH-oxidase 2 (NOX-2) in the cardiac bioenergetics dysfunction and oxidative stress encountered in T2D is measured in NOX-2 deficient mice and high fat-fed mice subjected to NOX-2 pharmacological inhibition. The authors show that NOX-2 suppression and inhibition improve cardiac function and decrease oxidative stress. A link between NOX-2, the activity of which consists of producing superoxide anions, and mitochondrial reactive oxygen species, is even highlighted.

In a second set of studies, several publications describe the effects of different interventions on systemic and cardiac dysfunction in the context of diabetes mellitus. Exercise training is known to protect the heart during T2D. In their study, Kar et al. [12] show that this beneficial effect is related to a change in the antioxidant hydrogen sulfide in the heart and a modulation of pyroptosis. A second study [13] describes the effects of the antioxidant alpha-lipoic acid given as chronic discontinuous treatment on several markers of insulin resistance, plasma oxidative stress and inflammation. The investigators reveal that the type of treatment given has beneficial actions on several of the studied parameters. A third study [14] deals with the effects of two well-known antioxidants, rosmarinic acid and sinapic acid, on several serum and cardiac oxidative stress markers in female diabetic rats. This study shows that both antioxidants given at adequate doses improve the markers of cardiac oxidative stress. Finally, a fourth study [15] reveals the influence of dietary eicosapentaenoic (EPA) acid on rat survival during diabetes mellitus and the effect of the association between this anti-inflammatory and antioxidant omega-3 polyunsaturated fatty acid in association with green tea extract (GTE). Surprisingly, EPA is lethal in diabetic rats and GTE completely alleviates this detrimental effect, suggesting that the choice of an adequate antioxidant is crucial to improve the health of diabetic patients.

The session is concluded by a review article [16] dealing with chronic kidney disease (CKD), which is known to be associated with strong mortality and a high risk of cardiovascular disease. Both animal and human models are reviewed in order to finally conclude that the redox balance is strongly involved in the detrimental consequences of CKD and that adequate antioxidant treatments might be beneficial.

If this Special Issue supplies important information on how to fight T2D-related oxidative stress, it does not explain the transition between moderate obesity, characterized by improved oxidative stress, mitochondrial function and cardiac mechanical activity, and the deterioration of cardiac function during diabetic cardiomyopathy. The resolution of this surprising paradigm may offer new lines of attack to prevent the development of T2D-related heart failure.
